# Impact of anemia on acute ischemic stroke outcomes: A systematic review of the literature

**DOI:** 10.1371/journal.pone.0280025

**Published:** 2023-01-05

**Authors:** Ansh Desai, David Oh, Elizabeth M. Rao, Saswat Sahoo, Uma V. Mahajan, Collin M. Labak, Rohit Mauria, Varun S. Shah, Quang Nguyen, Eric Z. Herring, Theresa Elder, Amber Stout, Berje H. Shammassian

**Affiliations:** 1 Case Western Reserve School of Medicine, Cleveland, OH, United States of America; 2 Cleveland Clinic Lerner College of Medicine, Cleveland, OH, United States of America; 3 Department of Neurosurgery, University Hospitals Cleveland Medical Center, Cleveland, OH, United States of America; 4 Division of Neurocritical Care, Department Neurology, University of Miami Miller School of Medicine, Miami, Florida, United States of America; Mahidol University, Faculty of Tropical Medicine, THAILAND

## Abstract

**Introduction:**

Anemia has been reported in nearly 40% of acute ischemic stroke (AIS) patients and is linked to significant morbidity and disability. The presence of anemia is associated with worse outcomes in AIS, specifically in the presence of large vessel occlusion (LVO). An optimal hemoglobin (Hb) target specific to this pathology has not yet been established. The goal of this review is to systematically review literature that observes the association that exists between AIS outcomes and hemoglobin (Hb) levels.

**Methods:**

A systematic review was performed in accordance with guidelines for the Preferred Reporting Items for Systematic Review and Meta-Analysis (PRISMA) to identify studies from 2008–2022. The following inclusion and exclusion criteria were used: studies of adult patients with AIS; must describe outcomes with regard to Hb levels in AIS (not limited to LVO); must be written in English. The clinical variables extracted included Length of Stay (LOS), modified rankin score (mRS), Hb levels, and mortality.

**Results:**

A total of 1,154 studies were gathered, with 116 undergoing full text review. 31 studies were included in this review. The age of patients ranged from 61.4 to 77.8. The presence of anemia in AIS increased LOS by 1.7 days on average and these patients also have a 15.2% higher rate of mortality at one year, on average.

**Discussion:**

This data suggests that the contemporary thresholds for treating anemia in AIS patients may be inadequate because anemia is strongly associated with poor outcomes (e.g., mRS>2 or mortality) and increased LOS in AIS patients. The current generalized Hb threshold for transfusion (7 g/dL) is also used in AIS patients, however, a more aggressive transfusion parameter should be further explored based on these findings. Further studies are required to confirm these findings and to determine if a more liberal RBCT threshold will result in clinical benefits.

## Introduction

Acute ischemic stroke (AIS) constitutes the majority of cerebrovascular accidents (87%) and is one of the leading causes of mortality worldwide [1]. One comorbidity commonly associated with AIS is anemia, reported in as many as 40% of AIS patients [2]. The link between anemia and worse outcomes for AIS patients, specifically those with large vessel occlusion (LVO), may lie in reduced oxygen delivery, altered blood viscosity, and impaired cerebral autoregulation [2]. A possible association between anemia and neurological outcomes after an AIS would be note-worthy because anemia has shown minimal association with neurological function among other populations, such as those with neuromuscular diseases [3].

Despite the prevalence of anemia in AIS, neither the optimal target hemoglobin (Hb) nor a standardized threshold for red blood cell transfusion (RBCT) has been fully explored. Currently, a Hb level of 7 g/dL serves as the threshold for transfusion as a general rule in critical care practice, based on the TRICC trial, which has been extrapolated to other patient populations [4, 5]. However, studies among myocardial infarction patients have indicated that more liberal RBCT thresholds, such as transfusing at 10 g/dL Hb with a goal of Hb >11 g/dL, doesn’t significantly alter length of stay (LOS), 30-day hospital costs, among other outcomes [3]. Additionally, current recommendations suggest that in acute coronary syndrome, Hb transfusion parameters between 8 and 10 g/dl should be considered [6].

Given the identification of this potential gap in the guidelines, the goal of this study is to systematically review manuscripts that have observed the association between AIS outcomes, Hb levels, and RBCT.

## Methods

### Search strategy and selection criteria

A literature search was performed in accordance with the Preferred Reporting Items for Systematic Review and Meta-Analysis (PRISMA) guidelines. The following databases were queried by a medical librarian (AS): PubMed, Ovid/Medline, Cochrane, Web of Science, and Scopus. The PubMed database was searched using a combination of MeSH subject headings and keywords (using the [title/abstract] field tag) for the terms: “anemia,”, “anaemia”, “erythrocytes,” “red blood cells,” “hemoglobin”, and “Hb”. Using appropriate Boolean operators, these terms were combined with MeSH terms and keywords ([title/abstract]) synonymous with ischemic strokes and cerebrovascular events and then a keyword search for outcomes was added. Animal studies were filtered out and remaining references were limited to English language only. No limits were placed on dates of publication or publication types.

### Inclusion and exclusion criteria

All indexed articles were considered for inclusion in this study and screened by reading the title and abstract. Studies selected for full text review had the following inclusion criteria: retrospective or prospective study, randomized controlled trial, systematic review, or case series with at least 10 patients; must include adult patients with AIS; must describe outcomes with regard to anemia at presentation among patients with AIS; must be written in English language. AIS must be defined using clinical and radiographic criteria. Exclusion criteria were studies with pediatric populations; pregnant patients; adult patients with hemorrhagic strokes; reviews, editorials, case reports, and case series with < 10 patients.

### Data extraction

**Fig 1:**
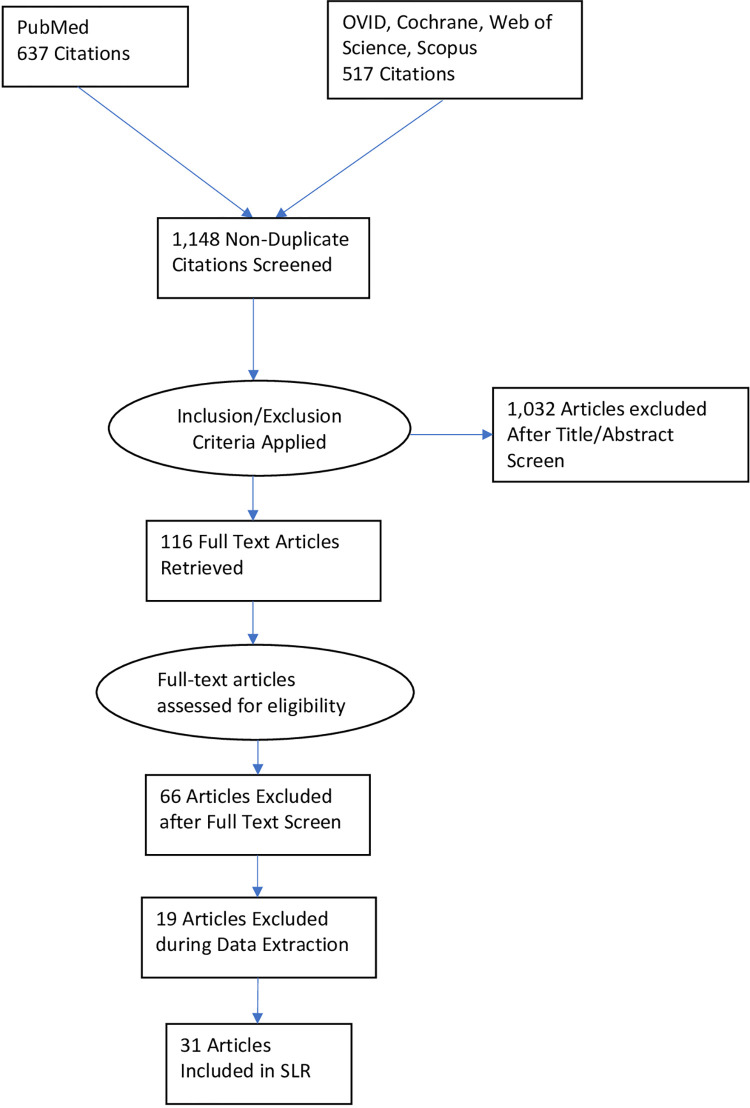
Summary PRISMA flow diagram of literature review.

Abstracts and full-text articles were screened by three reviewers (AD,DO,SS) and all conflicts were resolved by two arbiters (CML,EZH). Duplicate articles were removed during the screening process. Data was extracted and synthesized using Microsoft excel. Given the heterogeneity of data collection within the studies, only a qualitative synthesis was performed. The primary outcome measures were determined a priori and included length of stay (LOS), modified Rankin Score (mRS), and mortality. Length of stay (LOS) was defined as the number of days a patient remains in the hospital after admission and was consistently analyzed across the literature. Certain studies reported either only median or only mean LOS, so the values reported vary by study. The chosen outcomes attempted to provide a spectrum of measures spanning functional (mRS), health system (LOS), and clinical (mortality). Search results and article totals are shown in Fig 1.

### Quality assessment

The quality of the included studies was assessed using the Newcastle Ottawa Scale (NOS) by three reviews (AD, DO). This scale uses three categories (selection, comparability, and outcome) to assess the quality of studies included in systematic review. There are 3 possible points for selection, 2 points for Comparability (study controls for Age and NIHSS), and 3 points for Outcomes.

## Results

The literature search identified 1,154 publications, including 637 from PubMed and 517 from other sources. 6 were duplicates and removed. As a result, 1,148 unique articles were identified, and after abstract review, a total of 116 studies were selected for full-text review. Of these, 31 met inclusion criteria (Fig 1). Ten studies were prospective, 17 retrospective studies, 1 cohort, 2 cross-sectional, and 1 which included both prospective and retrospective components. Earliest study was published in 2008. The mean patient age studied ranged from 61.4 to 77.8 years (Table 1). LOS, mRS, and mortality were the three outcome measurements most consistently measured across the studies included in this review [7–23]. Anemia was defined using one of four parameters (Table 1). However, these parameters are not significantly different across studies and are thought to minimally impact the results of our study. The four parameters differ by no more than 1 g/dL. Furthermore, 58% of included studies used defined anemia as hemoglobin <13 g/dL for men or <12 g/dL for women. Hb was measured upon arrival across studies.

**Table 1:** Summary of included study demographics.

First Author Last Name	Country of Study	Study Type	Study total N	Mean age +/- SD	% Female	% of patients with Anemia at baseline^
Abe 2018*	Japan	Retrospective	480	69 +/- 13	33.7	N/A
Akpinar 2018*	Turkey	Retrospective	90	61 +/- 1.2	51.2	44.4
Alqahtani 2020 ***	USA	Retrospective	22,193	68 +/- 15	50.3	16.5
Barlas 2016*	United Kingdom	Retrospective	8,013	78 +/- 12	51.9	24.5
Barlas 2017*	United Kingdom	Retrospective	8,167	78 +/- 12	53.1	18.9
Bill 2013***	Switzerland	Cohort	243	70 +/- 17	53.5	N/A
Chan 2015**	Australia	Retrospective	103	70	59.0	28.2
Fabjan 2019*	Slovenia	Prospective	390	71 +/- 12	46.9	14.6
Furlan 2016 ****	Canada	Retrospective	9,230	N/A	48.6	28.8
Hao 2012 *	China	Prospective	1,176	N/A	42.7	29.8
Hatamian 2014 *****	Iran	Cross-Sectional	98	71 +/- 14	54.1	67.3
He 2020***	China	Prospective	326	65 +/- 10	36.2	19.0
Huang 2008*	Taiwan	Prospective	774	N/A	46.8	21.8
Huang 2008*	Taiwan	Prospective	66	68 +/- 11	22.4	28.8
Ke 2018**	China	Retrospective	32	N/A	72.0	71.9
Kellert 2011*	Germany	Retrospective	217	N/A	36.9	42.9
Kellert 2012*	Germany	Retrospective	100	70 +/- 13	40.0	19.0
Kellert 2015*	Germany	Prospective	691 (CAED patients)	N/A	42.4	6.90
Kellert 2014*	Germany	Retrospective	109	63 +/- 12	31.2	29.2
Kim 2019 ***	Korea	Retrospective	26	66 +/- 17	46.2	N/A
Kim 2020*	Korea	Retrospective	2,698	N/A	53.8	21.9
Kimberly 2011***	USA	Retrospective and Prospective	259	65 +/- 15	17.4	N/A
Li 2020 ***	China	Prospective	186	67 +/- 12	31.7	N/A
Liu 2020 ***	China	Prospective	8,303	66 +/- 8	62.5	N/A
Liu 2013*	China	Prospective	459	N/A	52.9	N/A
Luvizutto 2014***	Brazil	Cross-Sectional	40	66	37.5	N/A
Milionis 2015*	Switzerland	Retrospective	2,439	N/A	N/A	N/A
Pandian 2012*	India	Retrospective	449	58 +/- 14	N/A	46.7
Tanne 2010 *	Israel	Prospective	859	71 ± 13	42.2	N/A
Tseng 2018***	Taiwan	Retrospective	643	N/A	21.6	N/A
Sharma 2018*	USA	Retrospective	536	62 +/- 15	45.5	31.0

*anemia defined as hemoglobin <13 g/dL for men or <12 g/dL for women

**anemia defined as hemoglobin <120 g/L for men or <110 g/L for women

***anemia defined as hemoglobin <140 g/L for men or <120 g/L for women

**** anemia defined as hemoglobin <13.5 g/dL for men or <12 g/dL for women

^ anemia at baseline defined as anemia upon hospital admission

### Length of stay

Of the studies in our literature search which investigated the association between LOS and anemia, nearly all of them demonstrated that anemia is associated with a 1–3 day increase in LOS between anemic and normocythemic patients. One study showed a statistically significant difference between anemic and non-anemic ischemic stroke patients’ mean LOS (15.9d vs. 12.8d, p = 0.003) (Table 2) [24]. AIS patients with normochromic normocytic anemia were also more likely to have a LOS greater than 7 days (OR 1.21 [1.06–1.40]) [25]. Median LOS has also been shown to be higher for patients with greater declines in Hb (p < 0.001) [26]. The one conflicting study conducted by Kellert et al. saw LOS increase by 1 day for anemic vs. non-anemic patients, but this was not found to be a significant difference (p = 0.115) [19]. Furthermore, this was a smaller study consisting of 217 patients.

**Table 2:** Summary of included study demographics.

	Primary Outcome	Primary Author	Anemic	Non-anemic
**Mortality**	Death at 1 year (%)	Milionis (32)	31.1	14.4
		Tanne (39)	33.1	19.4
	Death/Disability at 1 year (%)	Tanne (39)	66.1	46.3
		Hao (33)	41.3	34.3
**Length of Stay**	Median (days)	Huang (15)	15.9	12.8
		Kellert (21)	7	6
		Milionis (32)	10	9
**Modified Rankin Score**	mRS > = 3 at 3 months (%)	He (17)	66.7	60.4
		Akpinar (2)	37.5	38

Common Outcomes Analyzed Across Selected Included Studies

### Modified rankin score

The studies included in this review demonstrate mixed results regarding the association between Hb level and mRS. One study found that patients with mRS 3–6 at discharge had significantly greater declines in Hb during their hospital stay (mean = 1.8 g/dL) compared to patients with mRS <3 at discharge (mean = 0.6 g/dL) [26]. A significant difference in admission Hb level between patients with mRS <2 vs. those with mRS >2 at discharge has also been shown (14.2 g/dL vs 10.9 g/dL, p = 0.002) [27]. Similarly, at 6-month follow up, patients with mRS 3–6 at 6 months post-stroke were more likely to have been anemic during their hospital stay compared to those with mRS 0–2 (56.2% vs 35.6%, p < 0.001) [28].

Conversely, some studies did not find the association between Hb and mRS to be significant. One study found that the difference in the frequency of number of patients with mRS 3–6 at discharge was not statistically significant between anemic (defined in the study as <12 g/dL for women and <13 g/dL for men) and non-anemic patients (37.5% vs 38%, respectively, p = 0.08) (Table 2) [2]. However, this same study found that when stratifying anemia by severity, patients in the severe anemia group (Hb < 8 g/dL) had poorer functional outcomes when compared with patients with less severe anemia (p = 0.003). Low Hb on admission (<14 g/dL for men and <12 g/dL for women) was not found to be an independent predictor of having an mRS 3–6 at discharge (OR = 1.09, 95% CI = 0.98–1.22, p = 0.12) following AIS [29].

There is substantial heterogeneity in the way that the above outcomes were measured and reported. For example, Kim et al. and Liu et al. both reported a significant difference in admission Hb levels and mRS. However, the duration of follow-up differed (6 months vs. 3 months, respectively.) In addition, Akpinar et al. evaluated patients of NIHSS ≥ 20 while Liu et al evaluated patients of NIHSS > 4.

### Mortality

Differences in mortality were consistently compared between anemic and non-anemic patients in the included studies. Overall, the long-term survival curves were significantly different between AIS patients with anemia vs. without anemia at presentation (Log-rank test p<0.001), with 3-year mortality rate being significantly higher in the anemia population (p = 0.021) [30]. Similarly, after adjusting for known risk factors of mortality post-AIS, such as NIHSS and age, anemia was shown to be an independent predictor of long-term morality (HR = 1.718, 95% CI = 1.055–2.800, p = 0.03) [31]. The mortality rate was 77.2% for anemic patients and 44.1% for non-anemic patients (p = 0.001).

This trend is true in shorter time periods as well. Anemia at presentation has been associated with increased mortality up to 1 year after admission [25]. Specifically at 7 days, the mortality rate was 15.2% for anemic patients and 6.7% for non-anemic patients; at 1-month, it was mortality was 23.9% for anemic patients and 13.2% for non-anemic patients; at 3 months, it was 24.1% for anemic and 10.7% for non-anemic; at 12 months, it was 31.1% for anemic and 14.4% for non-anemic (Table 2) [32]. Data from Hao et al. also indicated that the 1-year mortality was 25.4% for anemic and 16.5% for non-anemic (p = 0.001) (Table 2) [33]. It is noteworthy that Table 2 has a limited number of studies because mortality, length of stay, and mRS were not consistently compared or defined across studies.

However, the association between anemia and mortality is nuanced in this patient population, as decreases in Hb during hospital stay were also associated with increased mortality. As an example, data from Kellert et al. 2009 indicated that mortality after 3 months was associated with decreases in Hb (p = 0.04) and hematocrit (Hct) (p = 0.027) during hospital stay [34].

### Red blood cell transfusion

Based on the studies included in this review, it appears that outcomes following RBCT are dependent on timing and severity of anemia. One study by Kim et al. compared early (<48 hours) and late (>48 hours) RBCT after admission and found that patients who received later transfusions were associated with worse outcomes, defined as mRS>2 (p = 0.009). With regards to severity of anemia, another study found that RBCT when given with the general guideline of maintaining Hb in the range of 8 to 10 g/dL was associated with increased LOS (p<0.001) and mechanical ventilation (p<0.001). However, they did not find a significant association between RBCT and other outcomes such as in-hospital mortality and mRS at 90 days. Furthermore, Sharma et al. also noted an association between RBCT and poorer outcome when evaluating mRS at 3 months in univariate analysis. However, this association was not significant in the subsequent multivariate analysis. These findings suggest that RBCT does not have a significant association with worse outcomes and that earlier transfusion may improve outcomes.

### Study quality and risk of bias

NOS scores for all included studies were calculated (Table 3). Scores ranged from 5 to 9, with an average of 7.97. For the comparability category, studies were given points based on if they controlled for age and/or NIHSS.

**Table 3:** Risk of bias assessment.

First Author Last Name	Selection	Comparability (Study controls for 1. Age, 2. NIHSS)	Outcome	Total
Abe 2018 [8]	4	2	3	9
Akpinar 2018 [2]	4	2	3	9
Alqahtani 2020 [5]	2	1	3	6
Barlas 2016 [7]	4	1	3	8
Barlas 2017 [24]	3	1	3	7
Bill 2013 [11]	3	1	3	7
Chan 2015 [25]	4	0	3	7
Fabjan 2019 [14]	4	2	3	9
Furlan 2016 [12]	4	1	3	8
Hao 2012 [17]	4	2	3	9
Hatamian 2014 [26]	4	0	3	7
He 2020 [27]	4	2	3	9
Huang 2008 [6]	4	0	3	7

## Discussion

Given their impact on oxygen delivery and blood viscosity, Hb levels are likely to impact outcomes in AIS. However, there is no clearly delineated recommendation to optimize Hb parameters in AIS patients in the acute period. Current updates to the 2018 AHA guidelines for acute AIS management recommend that 1. Hemodilution is not beneficial, and 2. high-dose albumin is not beneficial. However, these guidelines fail to provide further insight on specific Hb goals in the AIS population. Furthermore, a 2021 study by Zhang and colleagues describe a U-shaped curve with regard to the relationship between Hb and mortality and poor functional outcome, suggesting that both anemia and polycythemia alike are detrimental in morbidity and mortality following AIS. Interestingly, the trough of their U-shaped models was located at a Hb of 15–16 g/dL for both parameters. They ultimately conclude that abnormal hemoglobin was associated with an increased risk of all‐cause mortality, poor functional outcome, stroke recurrence, and vascular events.

Most institutions utilize current Hb thresholds for RBCT (7 g/dL) that have been extrapolated from the Transfusion Requirements in Critical Care (TRICC) trial initially published in 1999. This has been suggested as a reasonable parameter in the neuro-critical care literature as well [35]. The more conservative RBCT parameter of 7 g/dL avoids known adverse associations with transfusion including worse neurocognitive outcomes at 1-year follow-up and the more acute risk of transfusion reaction [36, 37]. Despite this commonly used strategy, recent guidelines published by the British Committee in Hematology argue that among patients who have had a neurologic injury (e.g., aneurysmal subarachnoid hemorrhage, traumatic brain injury) and are hemodynamically stable, Hb should be maintained at 9 g/dL [38].

The results of this review demonstrate a difference in outcomes for AIS patients who develop anemia during hospital stay compared to those who do not. Being anemic increased an AIS patient’s LOS by a total of 1–3 days. Similarly, being anemic increased the chance of mortality at one year by 8.9%-16.7% [32, 33, 39]. Given that more conservative RBCT parameters (7 g/dL) increase the risk for anemia, such trends bring into question whether more lenient RBCT thresholds may be warranted among AIS patients. However, it must be appreciated that factors outside of anemia management have also been shown to impact LOS among AIS patients. For instance, older age (> 65 years of age), atrial fibrillation, history of smoking, among several other factors have also been shown to affect LOS in this patient population [40]. We are unable to control for these factors given the variation across studies, and this should be taken into account when appreciating the impact of anemia on LOS should.

We also find that there is a less clear association between anemia and mRS. Although some of the included studies on mRS outcome did not find a significant association between anemia and mRS, it is important to note that such studies used higher cutoffs for anemia (Furlan et al.) or found an association when stratifying anemia by severity (Akpinar et al.) Additionally, the association for mRS was likely not as evident given the neurologic injury that resulted from the stroke among both patient populations. In summary, our findings suggest that the contemporary thresholds for treating anemia in AIS patients can benefit from further investigations and evaluating more targeted RBCT thresholds.

However, the management of AIS is a multifactorial endeavor and correction of anemia alone will certainly not facilitate drastic improvements in patient outcomes. For instance, stroke recurrence and diabetes mellitus are two other factors that have been known to extend LOS in this patient population [40]. As a result, medical management of these entities should also be optimized. Similarly, poststroke BP management must continue to be emphasized, as higher systolic BP have been associated with poorer neurological function [41]. In other words, the proper management of anemia is certainly valuable in the setting of an AIS, but to truly improve outcomes, many other factors must be considered as well.

## Limitations

These results should be considered in light of the limitations present in this study. First, inclusion criteria for AIS patients in each study were heterogeneous. Akpinar et al. only included patients with NIHSS ≥ 20 while Liu et al. included patients with NIHSS > 4. These differences in stroke severity distribution inevitably contribute to observed mRS differences at follow up, regardless of timing. Second, several of the included studies took place internationally and included homogeneous demographic participants, potentially limiting generalizability. Additionally, the follow up time for outcome measures significantly varied across studies, with certain studies reporting mortality at one month, while others determined mortality only at one year. Such variation prevented consistent representation of outcomes across the studies included.

## Conclusions

Despite the heterogeneity of data presented in this review, we observe general trends toward increased LOS, mRS, and mortality in the presence of anemia in AIS. Further investigation is required to determine whether more liberal RBCT thresholds would be more beneficial than harmful in the AIS population as is observed in other hypoperfusive pathologies (e.g., non-ST elevation myocardial infarction).

## Supporting information

S1 Text Prisma checklist. (DOCX)
